# Correlations between pain in the back and neck/upper limb in the European Working Conditions Survey

**DOI:** 10.1186/s12891-019-2404-8

**Published:** 2019-01-23

**Authors:** Emanuele Rizzello, Georgia Ntani, David Coggon

**Affiliations:** 1 Department of Medical and Surgical Sciences (DIMEC), University of Bologna, Bologna, Italy; 2 Medical Research Council Lifecourse Epidemiology Unit, University of Southampton, Southampton, SO16 6YD UK; 3 Arthritis Research UK/MRC Centre for Musculoskeletal Health and Work, University of Southampton, Southampton, UK

**Keywords:** Low back pain, Upper limb pain, Prevalence, International variation

## Abstract

**Background:**

Recent research has suggested that wide international variation in the prevalence of disabling regional pain among working populations is driven largely by factors predisposing to musculoskeletal pain in general and not specific to individual anatomical sites. We sought to confirm this finding, using data from an independent source.

**Methods:**

Using data from the fifth (2010) and sixth (2015) European Working Conditions Surveys, we explored correlations between the one-year prevalence of pain in the back and neck/upper limb among people of working age across 33 European countries, and between changes in pain prevalence at the two anatomical sites from 2010 to 2015.

**Results:**

Each survey recruited ≥1000 participants per country, response rates ranging from 11 to 78%. In 2010, the estimated one-year population prevalence of back pain ranged from 23% in Ireland to 66% in Portugal, and that of pain in the neck/upper limb from 25% in Ireland to 69% in Finland, the prevalence of pain at the two anatomical sites being correlated across the 33 countries (*r* = 0.42). A similar pattern was apparent in 2015. For back pain, the percentage change in prevalence from 2010 to 2015 varied from − 41.4% (Hungary) to + 29.6% (Ireland), with a mean across countries of − 3.0%. For neck/upper limb pain, the variation was from − 41.0% (Hungary) to + 44.1% (Romania), with an average of − 0.1%. There was a strong correlation across countries in the change in pain prevalence at the two anatomical sites (*r* = 0.85).

**Conclusions:**

Our findings accord with the hypothesis that international variation in common pain complaints is importantly driven by factors that predispose to musculoskeletal pain in general.

## Background

The Cultural and Psychosocial Influences on Disability (CUPID) study has demonstrated wide international variation in the prevalence of disabling regional pain among working populations [1–3]. This appeared to be driven largely by unidentified causes that predispose to musculoskeletal pain in general rather than being specific to only one or two anatomical sites [2, 3]. Thus, across 45 occupational groups from 18 countries, the prevalence of disabling low back pain (LBP) correlated with the mean number of anatomical sites other than low back that had earlier been reported as painful [2]. And after allowance for occupation and potentially confounding psychosocial risk factors, individual risk of disability and sickness absence from LBP was predicted by the number of other anatomical sites which the person had previously reported as painful [2, 4]. Moreover, similar associations were apparent for pain in the wrist/hand [3].

If major international differences in disabling musculoskeletal pain are truly a consequence of factors promoting musculoskeletal pain in general, there could be important implications for preventive strategies. It would suggest a need to look beyond conventional ergonomic approaches, which tend to focus on localised mechanical loading of structures such as the spine or wrist/hand. Thus, it would be helpful to know whether correlations between the prevalence of pain in different bodily regions are apparent in other datasets covering multiple countries. The European Working Conditions Survey (EWCS) [5, 6] offered an opportunity to examine this for pain in the low back and neck/upper limb, and also to explore whether there were correlations across countries in the extent to which the prevalence of pain at these anatomical sites changed over time.

We therefore analysed data from two successive rounds of the European Working Conditions Survey (EWCS) [5, 6], aiming to assess: a) differences between countries in the prevalence of pain in the back and neck/upper limb at each of two time points; b) differences between countries in changes over time in the prevalence of back and neck/upper limb pain; and c) the extent to which prevalence rates and changes in prevalence for the two anatomical regions correlated across countries.

## Methods

The EWCS is a periodic survey conducted by the European Foundation for the Improvement of Living and Working Conditions (Eurofound) to provide information about the occupational circumstances and health of employees and self-employed workers across Europe. Detailed descriptions of its design and methods have been published elsewhere [5, 6].

We used data from the fifth and sixth surveys, which were conducted during January to August 2010, and February to December 2015. In each survey, the target population was all residents of participating countries, who were aged 15 years or older (16 or older in Spain, the UK and Norway) and currently in employment. Those eligible to take part were sampled with stratification by geographical region and level of urbanisation, either from population or address registers, or by a random route method with a screening procedure to select the eligible respondent within each household. Information was collected through face-to-face interviews, using a standardised questionnaire, which had been drafted originally in English and then translated into local languages (with checks for accuracy by independent back-translation). The subject matter was wide-ranging, but included two questions on experience in the past year of pain in the back and neck/upper limb (“Over the last 12 months did you have any of the following health problems … C - backache … D - muscular pains in shoulders, neck and/or upper limbs (arms, elbows, wrists, hands etc.)?”).

To render them more representative of the intended target population, prevalence estimates for each country had been corrected by Eurofound for differences in selection probabilities that were inherent in the sampling strategy, and also by post-stratification weighting for sex, age, region, occupation and sector of economic activity to allow for differences in willingness and ability to take part in the survey [7, 8].

Statistical analysis was carried out with Stata v.12.1 software (Stata Corp LP 2012, Stata Statistical Software: Release 12.1, College Station TX, USA). We focused on the 33 countries for which data were available from both surveys. Spearman rank correlation coefficients (r) were used to summarise correlations across countries between the prevalence of pain in the back and in the neck/upper limb, and between changes over time in the prevalence of pain at the two sites. The approximate statistical significance of correlation coefficients was determined based on reported total sample sizes by country, but without adjustment for the stratification that was applied in sampling and the use of post stratification weighting (on which we did not have sufficient data).

The data that we accessed from the EWCS surveys were retrospective anonymized summary statistics, and ethical approval was not therefore required.

## Results

Each survey recruited at least 1000 individuals per country (Table 1). In 2010, interviews were completed by 42,798 participants, with an overall response rate of 44% (ranging from 31% in Spain to 74% in Latvia). In 2015, 41,811 participants answered the questionnaire, the response rate varying from 11% in Sweden to 78% in Albania, with an average across all countries of 43%.

**Table 1:** Response rates and prevalence of musculoskeletal pain by country and year of survey

Country	2010				2015			
	Number interviewed	Response rate (%)	One-year prevalence of pain (%)		Number interviewed	Response rate	One-year prevalence of pain (%)	
			Back	Neck-upper limb			Back	Neck-upper limb
Albania (AL)	1000	58	39	43	1002	78	31	39
Austria (AT)	1003	32	46	43	1028	47	47	44
Belgium (BE)	4001	34	44	40	2587	36	46	45
Bulgaria (BG)	1014	66	34	33	1064	64	38	41
Croatia (CR)	1100	43	49	46	1012	50	50	51
Cyprus (CY)	1000	66	46	45	1003	69	45	49
Czech Republic (CZ)	1000	47	55	44	1002	63	40	31
Denmark (DK)	1069	58	40	50	1002	26	45	58
Estonia (EE)	1000	56	56	61	1015	59	48	57
Finland (FI)	1028	47	50	69	1001	33	48	69
France (FR)	3046	34	53	50	1527	37	60	57
FYROM^a^	1100	68	45	49	1011	75	43	42
Germany (DE)	2133	56	51	43	2093	51	42	35
Greece (GR)	1037	40	43	39	1007	64	38	39
Hungary (HU)	1006	47	47	50	1023	58	27	30
Ireland (IE)	1003	50	23	25	1057	54	30	30
Italy (IT)	1500	34	51	48	1402	61	41	35
Latvia (LV)	1001	74	58	53	1004	62	51	46
Lithuania (LT)	1004	54	52	39	1004	62	51	40
Luxembourg (LU)	1000	40	43	42	1003	43	53	50
Malta (MT)	1000	52	45	37	1004	46	41	43
Montenegro (ME)	1041	59	49	46	1005	71	48	40
Netherlands (NL)	1017	37	36	43	1028	37	37	41
Norway (NO)	1085	32	41	53	1028	51	40	51
Poland (PL)	1500	44	46	40	1203	56	47	42
Portugal (PT)	1000	44	66	56	1037	55	42	39
Romania (RO)	1017	59	48	37	1063	55	57	53
Slovakia (SK)	1002	57	54	39	1000	65	50	36
Slovenia (SI)	1404	42	53	49	1607	47	48	42
Spain (ES)	1008	31	44	43	3364	32	46	45
Sweden (SE)	1004	35	39	52	1002	11	41	54
Turkey (TR)	2100	56	41	47	2000	36	46	49
United Kingdom (UK)	1575	37	34	33	1623	42	35	37
All countries	42,798	44	46	44	41,811	43	45	44

^a^Former Yugoslav Republic of Macedonia

In 2010, the one-year prevalence of back pain ranged from 23% in Ireland to 66% in Portugal, while that of pain in the neck/upper limb varied from 25% in Ireland to 69% in Finland (Table 1). Moreover, the prevalence of pain at the two anatomical sites was correlated across the 33 countries (*r* = 0.42, *p* = 0.015). A similar pattern was apparent in 2015. The prevalence of back pain ranged from 27% (Hungary) to 60% (France), and that of pain in the neck/upper limb from 30% (Hungary) to 69% (Finland), with a correlation coefficient of 0.56 (*p* = 0.001).

**Fig. 1:**
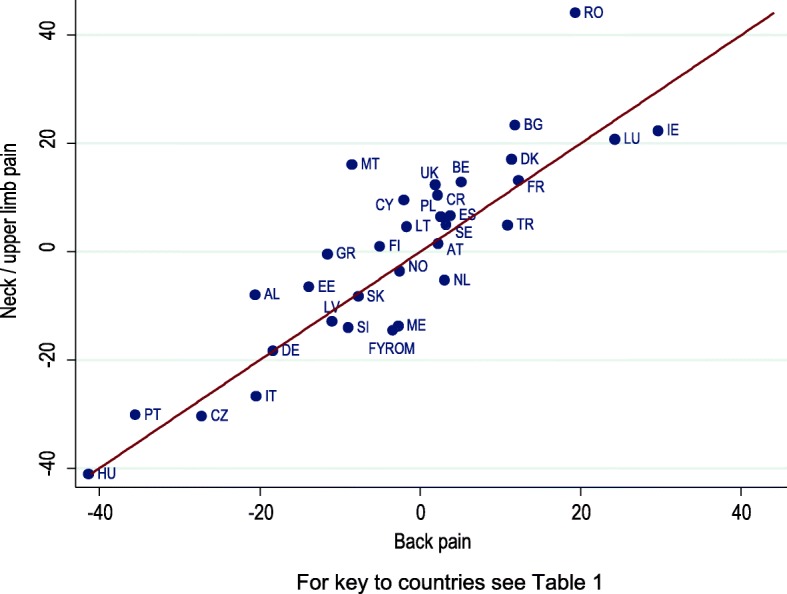
Percentage change from 2010 to 2015 in one-year prevalence of back and neck/upper limb pain by country

Figure 1 plots the percentage change in the one-year prevalence of neck/upper limb pain from 2010 to 2015 against that for back pain. For back pain, the percentage change varied from − 41.4% (Hungary) to + 29.6% (Ireland), with a mean across countries of − 3.0%. For neck/upper limb pain, the variation was from − 41.0% (Hungary) to + 44.1% (Romania), with an average of − 0.1%. Again, there was a strong correlation across countries (*r* = 0.85, *p* < 0.001).

## Discussion

Our analysis shows moderate to strong correlations across 33 European countries between the prevalence of reported pain in the back and neck/upper limb, and between changes over time in the prevalence of pain in the two anatomical regions. These findings are consistent with the hypothesis that international variation in common pain complaints is importantly driven by factors that predispose to musculoskeletal pain in general.

The investigation was based on more than 1000 participants per survey in each participating country, and the large differences in the prevalence of symptoms between countries (more than two-fold), and within countries over time (up to 40%), are unlikely to have occurred simply by chance. For example, in a sample of 1000, the 95% confidence interval around a prevalence of 40% would be 37 to 43%.

The average response rate to the surveys was less than 50%, and that in Sweden in 2015 was as low as 11%. Adjustments were made for differences in participation by sex, age, region, occupation and sector of economic activity. Moreover, the questions about pain were only a small component of a wide-ranging questionnaire. Nevertheless, it is possible that those who took part were unrepresentative in their experience of pain. There was no systematic difference in the prevalence of pain by response rate (overall correlation coefficients across all countries and both surveys = − 0.02, *p* = 0.89 for back pain and − 0.30 *p* = 0.015 for neck/upper limb pain), but for both anatomical regions, within-country changes in prevalence from 2010 to 2015 correlated with changes in response rate between the two surveys (*r* = − 0.42, *p* = 0.014 for back pain and *r* = − 0.43, *p* = 0.013 for neck/upper limb pain). Thus, while within-survey correlations in the prevalence of pain at the two anatomical sites are unlikely to have been influenced by differences in response rate, the correlation between changes over time in the prevalence of pain at the two sites may have been somewhat inflated.

Another possible source of bias might be differences in understanding of terms for pain when questionnaires were translated into local languages. However, that could not account for the changes in prevalence that were observed within countries over time, or for the strong correlation in such changes between pain in two distinct anatomical regions.

It could be that pain in the neck/shoulder renders people more prone to pain also in the back, or vice versa. For example, pain in one region might cause individuals to modify their postures or activities in a way that predisposes to pain elsewhere, or it might make them more aware of, and willing to report pain at other sites. We do not know what proportion of participants in the EWCS surveys reported pain in both the back and neck/upper limb, but in the CUPID study, multisite pain was common [9].

Alternatively, the observed correlations could reflect the effects of shared causes for pain at multiple anatomical sites. Our analysis does not indicate what those causes might be, but the relationship between pain at different sites in the CUPID study was present in people carrying out similar occupational activities, and after adjustment for established risk factors such as low mood, somatising tendency, fear-avoidance beliefs and psychosocial aspects of work [2, 3]. The shared causes could be unrecognised biomechanical factors, or perhaps more likely, physiological or psychological determinants of pain perception.

Whatever the nature of the causes, our results suggest that their impact can change importantly over time periods as short as five years, and that it has been going up in some European countries (e.g. Ireland, Romania and Luxembourg) while declining in others (e.g. Portugal, Czech Republic and Italy). If so, they may be amenable to preventive interventions, with potentially major benefits. Research is now needed to identify and characterise those unidentified causes, focusing on factors that could predispose to musculoskeletal pain in general rather than being site-specific.

## Conclusions

Our findings provide independent corroboration that major differences in the prevalence of musculoskeletal pain in different anatomical regions correlate across countries. As such, they support the hypothesis that international variation in common pain complaints is importantly driven by factors that predispose to musculoskeletal pain in general.

## Abbreviations

CUPID: Cultural and Psychosocial Influences on Disability
Eurofound: European Foundation for the Improvement of Living and Working Conditions
EWCS: European Working Conditions Survey
LBP: Low back pain
r: Spearman rank correlation coefficient
